# Expression, Purification, and Characterization of *Plasmodium vivax* Lactate Dehydrogenase from Bacteria without Codon Optimization

**DOI:** 10.3390/ijms241311083

**Published:** 2023-07-04

**Authors:** Yeon-Jun Kim, Jun-Seop Shin, Kang Woo Lee, Hyo-Ji Eom, Byung Gwan Jo, Jin Woo Lee, Jun Hyoung Kim, So Yeon Kim, Jung Hoon Kang, Jae-Won Choi

**Affiliations:** 1 Department of Biomedical Science, Cheongju University, Cheongju 28160, Republic of Korea; 2 College of Pharmacy, Duksung Women’s University, Seoul 01369, Republic of Korea; 3 Division of Infectious Diseases, Department of Internal Medicine, Chungbuk National University Hospital, Cheongju 28644, Republic of Korea; 4 Department of Dental Hygiene, Cheongju University, Cheongju 28503, Republic of Korea; 5 Department of Biopharmaceutical Sciences, Cheongju University, Cheongju 28160, Republic of Korea

**Keywords:** malaria, *Plasmodium vivax*, lactate dehydrogenase, Rosetta(DE3)

## Abstract

*Plasmodium vivax* is the most widespread cause of malaria, especially in subtropical and temperate regions such as Asia-Pacific and America. *P. vivax* lactate dehydrogenase (PvLDH), an essential enzyme in the glycolytic pathway, is required for the development and reproduction of the parasite. Thus, LDH from these parasites has garnered attention as a diagnostic biomarker for malaria and as a potential molecular target for developing antimalarial drugs. In this study, we prepared a transformed *Escherichia coli* strain for the overexpression of PvLDH without codon optimization. We introduced this recombinant plasmid DNA prepared by insertion of the *PvLDH* gene in the pET-21a(+) expression vector, into the Rosetta(DE3), an *E. coli* strain suitable for eukaryotic protein expression. The time, temperature, and inducer concentration for PvLDH expression from this *E. coli* Rosetta(DE3), containing the original *PvLDH* gene, were optimized. We obtained PvLDH with a 31.0 mg/L yield and high purity (>95%) from this Rosetta(DE3) strain. The purified protein was characterized structurally and functionally. The PvLDH expressed and purified from transformed bacteria without codon optimization was successfully demonstrated to exhibit its potential tetramer structure and enzyme activity. These findings are expected to provide valuable insights for research on infectious diseases, metabolism, diagnostics, and therapeutics for malaria caused by *P. vivax*.

## 1. Introduction

Malaria is a febrile, infectious disease caused by *Plasmodium* parasites transmitted through the bite of female *Anopheles* mosquitoes, which mediate its entry into the human body [1]. Globally, more than 200 million cases of malaria occur causing approximately 600,000 deaths annually [2]. The *Plasmodium* parasites have a complex life cycle marked by successive rounds of asexual replication across various stages and tissues, both in the intermediate vertebrate host and in the definitive insect host: when they infect humans through a mosquito bite, they move to the liver and/or red blood cells, and chiefly reproduce asexually; and in their last stage of development in red blood cells they reproduce sexually [2,3,4]. Among the existing >100 species of *Plasmodium*, only five cause malaria in humans: *P. vivax*, *P. falciparum*, *P. ovale*, *P. malariae*, and *P. knowlesi* [1,5]. Symptoms of malaria generally include fever, headache, myalgia, nausea, and vomiting. In several cases, profound anemia, liver failure, acute kidney insufficiency, and diminished consciousness may occur [3,5,6].

Globally, *P. falciparum* and *P. vivax* account for the majority of cases of malaria. While *P. falciparum* is responsible for more deaths, *P. vivax* is the most widespread of all the malaria species and results in significant global morbidity and mortality [1]. *P. falciparum* is most common in tropical climates such as Africa, whereas *P. vivax* is distributed across tropical, subtropical, and temperate regions [1,7,8]. *P. vivax* forms a hypnozoite (dormant form) in the liver, which unless completely eliminated, can get re-activated and cause recurrence of malarial infection [9,10,11,12,13]. In addition, recent studies have revealed that *P. vivax* evades the host immune system [14], is fatal to infants and children [15], and invades the reticulocytes (called young red blood cells) [16,17,18]. Therefore, measures for eliminating *P. vivax* are completely warranted. Treatment with primaquine containing 8-aminoquinoline, which removes hypnozoites, can suppress the recurrence of malaria [19,20,21].

Although the blood smear method, which confirms the presence of *Plasmodium* parasites using light microscopy, is still the gold standard for diagnosing malaria [19,22,23], it has several disadvantages such as requiring several days for diagnosis and review by a pathologist. Diagnosis through polymerase chain reaction (PCR) using gene amplification is more sensitive than the blood smear method, requiring only a few hours [24,25], but with the disadvantage of requiring expensive laboratory equipment, resources, and various reagents. Therefore, the rapid antigen test based on antigen–antibody reaction detection, which quickly detects malaria on field without additional equipment or a pathologist, is widely used as the initial response to suspected malarial cases [19,26].

Representative biomarkers for the rapid diagnosis of malaria include lactate dehydrogenase (LDH) [27,28,29], histidine rich protein-2 (HRP-2) [30], glutamate dehydrogenase [31], aldolase [32], and merozoite surface protein [33,34]. Since only *P. falciparum* expresses HRP-2, it is not suitable as a biomarker for diagnosing malaria caused by *P. vivax*. In contrast, LDH is expressed in all *Plasmodium* species. It is overexpressed at the trophozoite stage in red blood cells during infection [19,27,28,29]. High LDH concentrations (219.1–1803.1 ng/mL) have been observed in the blood of patients with malaria [35]. LDH can be used to diagnose malaria through blood, rather than tissue, in suspected cases of infection. Thus, plasmodial LDH is a feasible biomarker for malarial diagnosis. The expression level of LDH among different *Plasmodium* species is highly similar. However, differences in the amino acid sequences between the species make it possible to distinguish infections caused by *P. vivax* from other species. In addition, plasmodial LDH is a crucial target for development of antimalarial drugs [19,20,21]. Therefore, studies on the effective production of *P. vivax* LDH (PvLDH) for diagnosing and developing therapeutic agents for malaria are required.

*P. vivax* is an eukaryote. Therefore, expressing and producing PvLDH using eukaryotic cells, such as mammalian or insect cells, may be advantageous. However, protein expression and production using eukaryotic cells have the disadvantages of consuming substantial time, labor, and cost. To overcome these disadvantages, a bacterial system is used for protein expression and purification [36,37]. To date, *E. coli* strains such as DH5α [38,39], JM105 [40], BL21(DE3) [41,42], and BL21(DE3)pLysS [43] have been used for the expression of PvLDH. While optimization of purification and expression of *P. falciparum* LDH has been widely reported, PvLDH has not received much attention in this regard. In this study, we used the Rosetta(DE3) strain, which is designed to enhance the expression of eukaryotic proteins that contain codons rarely used in *E. coli*. This strain supplies tRNA for AGA, AGG, AUA, CCC, CUA, and GGA codons, mainly used by eukaryotic cells for protein production [44,45]. The original sequence of *PvLDH* gene was introduced into Rosetta(DE3) without codon optimization. The temperature, time, and inducer concentration for PvLDH overexpression were optimized and the expressed PvLDH was purified. Furthermore, structural and functional properties of the purified PvLDH was studied based on the confirmation of tetramer formation and enzyme activity.

## 2. Results and Discussion

### 2.1. Construction of Recombinant Plasmid for Recombinant PvLDH Expression and Its Transformation into Bacteria for Protein Expression

**Figure 1:**
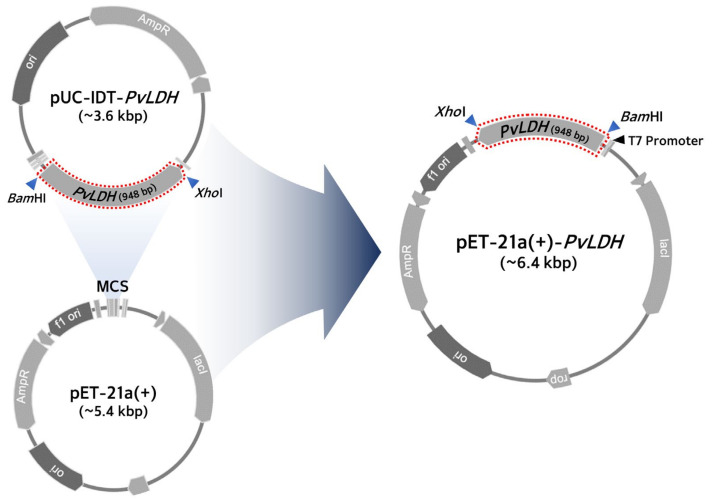
**Schematic diagram of the plasmid construction containing *PvLDH* cDNA.** Both the PCR product for *PvLDH* obtained from the pUC-IDT-*PvLDH* plasmid and pET-21a(+) were treated with *Bam*HI and *Xho*I to form sticky ends. The recombinant DNA was constructed using T4 ligase and *Bam*HI/*Xho*I restriction sites. The constructed plasmid was inserted into the Rosetta(DE3) strain for recombinant PvLDH expression. AmpR, ampicillin resistance gene; kbp, kilobase pair; lacI, lactose inhibitor; MCS, multiple cloning site; *PvLDH*, *Plasmodium vivax* lactate dehydrogenase coding gene.

The *PvLDH* gene was subcloned from pUC-IDT-*PvLDH* recombinant plasmid by inserting it into a pUC-type vector containing an ampicillin-resistance gene, and a large amount of plasmid was obtained by introducing the recombinant DNA into an *E. coli* DH5α strain which has a high plasmid copy number. In this study, the *PvLDH* gene was designed with a *Bam*HI restriction site at the 5′-end and *Xho*I restriction site at the 3′-end to construct the recombinant plasmid (Figure 1).

**Figure 2:**
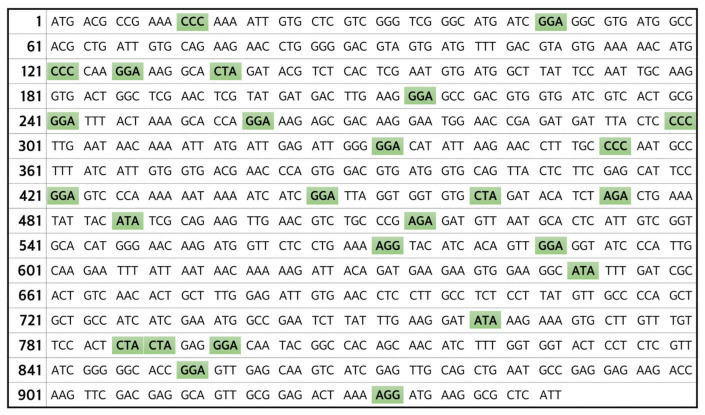
***PvLDH* cDNA sequence matches the bacterial rare codons.** The full-length sequence of the *PvLDH* cDNA (948 bp). Codons highlighted in green are those commonly used in eukaryotic cells but rarely used in bacteria. However, codons highlighted in green also indicate those that can be effectively translated into proteins using the Rosetta(DE3) strain, which is specialized for eukaryotic proteins, unlike other bacterial strains. *PvLDH*, *Plasmodium vivax* lactate dehydrogenase coding gene.

From this recombinant plasmid, the *PvLDH* gene was obtained via PCR for cloning, using specific forward and reverse primers for the *PvLDH* gene. First, for the overexpression of the target protein, we selected the pET-21a(+) vector, belonging to the pET series which contains a strong T7 promoter. For PvLDH protein expression, we inserted *PvLDH* gene in the pET-21a(+) vector appropriately in the multiple cloning site. The pET-21a(+)-*PvLDH* recombinant plasmid was successfully constructed by the restriction digestion of both the purified *PvLDH* gene DNA obtained from the PCR product and the pET-21a(+) plasmid with *Bam*HI and *Xho*I restriction endonucleases, followed by ligation with T4 DNA ligase (Figure 1). We transformed this recombinant plasmid by introducing it into an *E. coli* Rosetta(DE3) strain. There were two reasons for selecting this strain. First, since the Rosetta(DE3) expresses T7 RNA polymerase, it was advantageous to use a pET-21a(+) vector which contains the T7 promoter for enhancing the protein expression. Thus, the BL21 strain which does not express T7 polymerase was not considered for our study. Second, from the full length of the *PvLDH* gene of 948 bp (Figure 2), we identified 78 bp corresponding to 8.2% of the total length to have codons that are not preferred by *E. coli* strains such as DH5α, JM105, BL21(DE3), and BL21(DE3)pLysS. Hence, we used the Rosetta(DE3) strain, which is specially designed to enhance the expression of eukaryotic proteins contain codons rarely used in *E. coli*, and has tRNAs for all the codons of the *PvLDH* gene, including bacterial rare codons, such as AGA, AGG, AUA, CCC, CUA, and GGA [44,45], for the efficient translation of the *PvLDH* gene.

**Figure 3:**
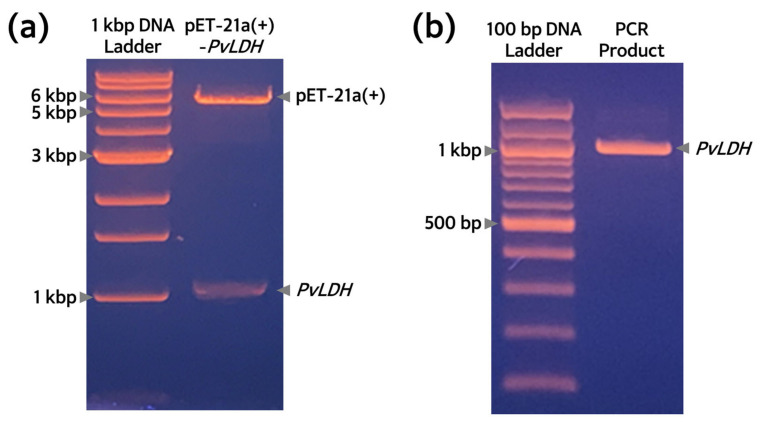
**Identification of pET-21a(+)-*PvLDH* plasmid from the transformed Rosetta(DE3) strain.** (**a**) Agarose gel image of plasmids extracted and purified from Rosetta(DE3) strain, and digested with restriction enzymes (*Bam*HI and *Xho*I). Gel electrophoresis was performed on a 0.7% agarose gel. (**b**) Agarose gel image of the PCR product using *PvLDH*-specific primers of the purified plasmid extracted from the Rosetta(DE3) strain. Gel electrophoresis was performed on an 1.0% agarose gel. kbp, kilobase pair; *PvLDH*, *Plasmodium vivax* lactate dehydrogenase coding gene.

The *E. coli* Rosetta(DE3) strain containing the pET-21a(+)-*PvLDH* plasmid was cultured and the plasmid DNA was purified and digested with *Bam*HI and *Xho*I restriction enzymes to confirm the insertion of the *PvLDH* gene into the vector. The digested plasmid DNA showed bands of ~5.4 kb and ~1.0 kb for pET-21a(+) and the inserted *PvLDH* gene (Figure 3a). Additionally, for confirmation of the insertion of the *PvLDH* gene, 1 ng recombinant plasmid was used as a template for PCR amplification of the *PvLDH* gene, with gene-specific primers, and a single band of ~1.0 kb was obtained (Figure 3b). Put together, these findings confirmed that pET-21a(+)-*PvLDH* had been successfully inserted into the Rosetta(DE3) strain. Furthermore, the inserted gene sequence was validated with automated DNA sequencing analysis using specific primers for the T7 promoter and terminator. Since sequencing of the *PvLDH* gene was successful with the use of these T7-specific primers, the successful construction of pET-21a(+)-*PvLDH* was confirmed.

### 2.2. Establishment of Optimal Conditions for PvLDH Overexpression, Isolation, and Purification

**Figure 4:**
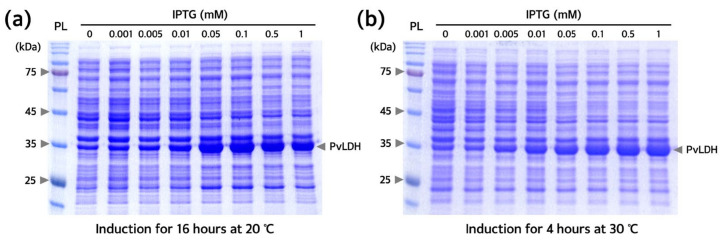
**Optimization of induction conditions for PvLDH overexpression.** (**a**) Sodium dodecyl sulfate-polyacrylamide gel electrophoresis (SDS-PAGE) image of the Rosetta(DE3) strain subjected to low-temperature induction at 20 °C for 16 h. (**b**) SDS-PAGE image of Rosetta(DE3) strain subjected to high-temperature induction at 30 °C for 4 h. Both experiments were conducted under the same conditions and the overexpression of PvLDH was induced using different IPTG concentrations. SDS-PAGE was performed on both gels using a 12% separating gel. Arrows on the right of each gel indicate overexpressed PvLDH. IPTG, isopropyl β-D-1-thiogalactopyranoside; kDa, kilodalton; PL, protein ladder; PvLDH, *Plasmodium vivax* lactate dehydrogenase.

**Figure 5:**
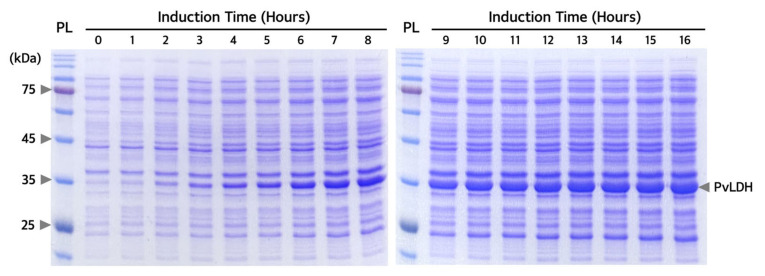
**Confirmation of overexpression pattern of PvLDH by induction time.** Sodium dodecyl sulfate-polyacrylamide gel electrophoresis (SDS-PAGE) image of Rosetta(DE3) strain treated with 0.1 mM IPTG and subjected to low-temperature induction at 20 °C for 16 h. The Rosetta(DE3) strain was collected at 1 h intervals, and SDS-PAGE was performed using a 12% separating gel. Arrows on the right side of the gel indicate overexpressed PvLDH. IPTG, isopropyl β-D-1-thiogalactopyranoside; kDa, kilodalton, PL, protein ladder; PvLDH, *Plasmodium vivax* lactate dehydrogenase.

Conditions for recombinant PvLDH overexpression were optimized for the transformed Rosetta(DE3) strain containing the pET-21a(+)-*PvLDH* plasmid. The expression pattern of PvLDH was confirmed by the appearance of a band of ~35 kDa, identified as PvLDH, which became thicker with increasing isopropyl β-D-1-thiogalactopyranoside (IPTG) concentration. Upon induction with 0.1 mM IPTG, PvLDH was sufficiently overexpressed (Figure 4). Thus, the overexpression of PvLDH in Rosetta(DE3) strain required a 10-times lower concentration of IPTG than that required (1 mM) for the overexpression of PvLDH in other strains such as DH5α, JM105, and BL21(DE3)pLysS strains [38,39,40,43]. Since IPTG is a relatively expensive reagent among those used for purification, its application at low concentrations is advantageous. Therefore, the method used in this study may reduce the cost for the mass production of PvLDH. PvLDH induction was achieved well at both low and high temperatures (20 °C and 30 °C), with a low temperature resulting in a slightly higher expression of PvLDH. Low-temperature expression is advantageous for protein solubility and stable protein production [36,37]. At 0.1 mM IPTG treatment and 20 °C, PvLDH was to be actively overexpressed even after 16 h induction (Figure 5). Thus, induction with 0.1 mM IPTG at 20 °C for 16 h was determined to be the optimal expression conditions for the purification of PvLDH.

**Figure 6:**
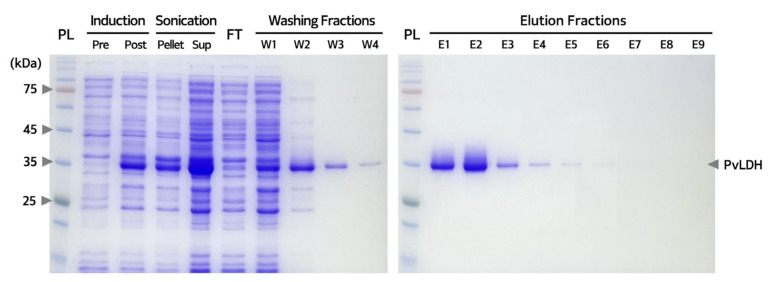
**Analysis of fractions obtained during PvLDH purification.** Sodium dodecyl sulfate-polyacrylamide gel electrophoresis (SDS-PAGE) images confirming the presence of PvLDH in pellets and supernatants (Sup) obtained from induced cells. FT represents the flow-through fraction generated from affinity chromatography using nickel-nitrilotriacetic acid (Ni-NTA). W1, W2, W3, and W4 refer to sequential washing fractions. E1–E9 represent the elution fractions generated from affinity chromatography using Ni-NTA. SDS-PAGE was performed on 12% separating gel. The arrow on the right side of the gel indicates PvLDH. kDa, kilodalton; PL, protein ladder; PvLDH, *P. vivax* lactate dehydrogenase; Pre, pre-induction; Post, post-induction; FT, flow-through fraction; W, washing fraction; E, elution fraction.

To purify the recombinant His-tagged PvLDH protein following overexpression using the Rosetta(DE3) strain under the established culture and induction conditions, the bacterial cultures following incubation were centrifuged, pellets were collected, dissolved in a lysis buffer and then lysed using an ultrasonic processor. A high amount of PvLDH was present in the supernatant obtained through additional centrifugation. Additionally, we confirmed that it could be obtained in soluble form (Figure 6). The soluble lysate was passed through a filtration column packed with nickel-nitrilotriacetic acid (Ni-NTA) resin to perform affinity chromatography using gravity flow. The successful purification of PvLDH was confirmed using sodium dodecyl sulfate-polyacrylamide gel electrophoresis (SDS-PAGE) of the fractions eluted from the initially loaded lysate; the fraction from the washing buffer that was passed through the column at 30-times more volume than the resin volume; and the fraction from the elution buffer was passed through the column at 10-times more volume than the resin volume (Figure 6). Finally, a washing buffer containing 50 mM imidazole was used to enhance the purity of PvLDH. Successfully obtained elution fractions were pooled together and subjected to two rounds of dialysis (using a cellulose membrane with a molecular weight cut-off of 6000–8000 Da to remove the unnecessary imidazole) for subsequent experiments and stored in phosphate-buffered saline (PBS). Purified PvLDH was quantified by bicinchoninic acid (BCA) protein assay and also verified by measuring absorbance at 280 nm.

We obtained 6.20 mg of PvLDH from 200 mL culture of Rosetta(DE3) strain. Alternatively, a PvLDH yield of 31.0 mg/L was obtained in this study. Several bacterial strains have been used for the purification of PvLDH. However, yield comparisons have not been reported as no specific yields were mentioned in previous reports. Our yield was approximately 1.72- to 2.07-times higher than PfLDH, which has high homology (>90%) with PvLDH, obtained from TG2 strain (15.0 mg/L) [46] and SG13009 (18.0 mg/L) [47]. Since the Rosetta(DE3) strain has tRNAs that carry bacterial rare codons that other strains do not have, in this study, we obtained a large amount of PvLDH without bacterial codon optimization. Above all, the production of a target protein using a bacterial system has may advantages compared to using an eukaryotic system [36,37,48]. First, a bacterial system is less labor-intensive because the time taken to obtain the target protein is short. Bacterial cells used for protein expression have a doubling time of less than 20 min, whereas most eukaryotic cells used for similar purposes have a doubling time of several hours to a day. For this reason, it takes 2 to 3 days from the culture to the overexpression and purification of a recombinant protein from bacterial cultures, whereas it takes 1 to 2 weeks when eukaryotic cells are used. Second, in terms of cost to obtain the target protein, the bacterial system is very economical, since they require only a simple undefined medium (like LB broth) and a shaking incubator, whereas eukaryotes (such as mammalian and insect cells) require a CO_2_ incubator (or bioreactor) and a defined medium containing serum or growth factors. Therefore, our proposed method for the production of PvLDH is advantageous.

### 2.3. Characterization of Purified PvLDH

**Figure 7:**
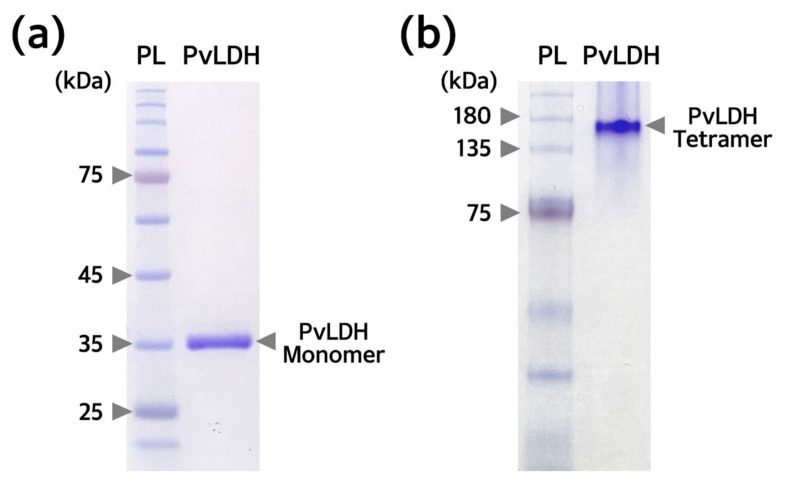
**Confirmation of PvLDH purity and tetramer formation by polyacrylamide gel electrophoresis.** (**a**) Confirmation of PvLDH using sodium dodecyl sulfate-polyacrylamide gel electrophoresis (SDS-PAGE) under reducing conditions. The polyacrylamide gel was electrophoresis using a 12% separating gel. PvLDH without impurities was detected at ~35 kDa, and was expected to be a monomer. (**b**) Confirmation of PvLDH using PAGE without SDS and a boiling step in native condition. PAGE was performed on the 10% separating gel. A band of ~140 kDa was located between the 135 kDa and the 180 kDa bands, indicating a tetramer structure of PvLDH without impurities. PvLDH (2 µg) was loaded onto both gels. kDa, kilodalton; PL, protein ladder; PvLDH, *P. vivax* lactate dehydrogenase.

The purity of the PvLDH protein obtained in this study was determined using SDS-PAGE under reducing and non-reducing conditions. Under reducing conditions, when PvLDH was stained with Coomassie Brilliant Blue R-250, a single band of ~35 kDa was obtained. This indicated the molecular weight of PvLDH monomer and the absence of other bands also indicated high purity (>95%) (Figure 7a). However, under non-reducing conditions, when PvLDH was stained with Coomassie Brilliant Blue R-250, a single band was identified between 135–180 kDa bands (Figure 7b). PvLDH has a pI value of 6.96, which is not located between Cl^−^ and glycine contained in the electrophoresis buffer on the separating gel. Thus, it moved according to its molecular weight and we speculated that PvLDH formed a tetramer and with a molecular weight of ~140 kDa. It is well established that LDH has a tendency to form tetramers. Therefore, it can be concluded that PvLDH, an eukaryotic protein synthesized from the Rosetta(DE3) strain, was normally folded into its native three-dimensional structure.

**Figure 8:**
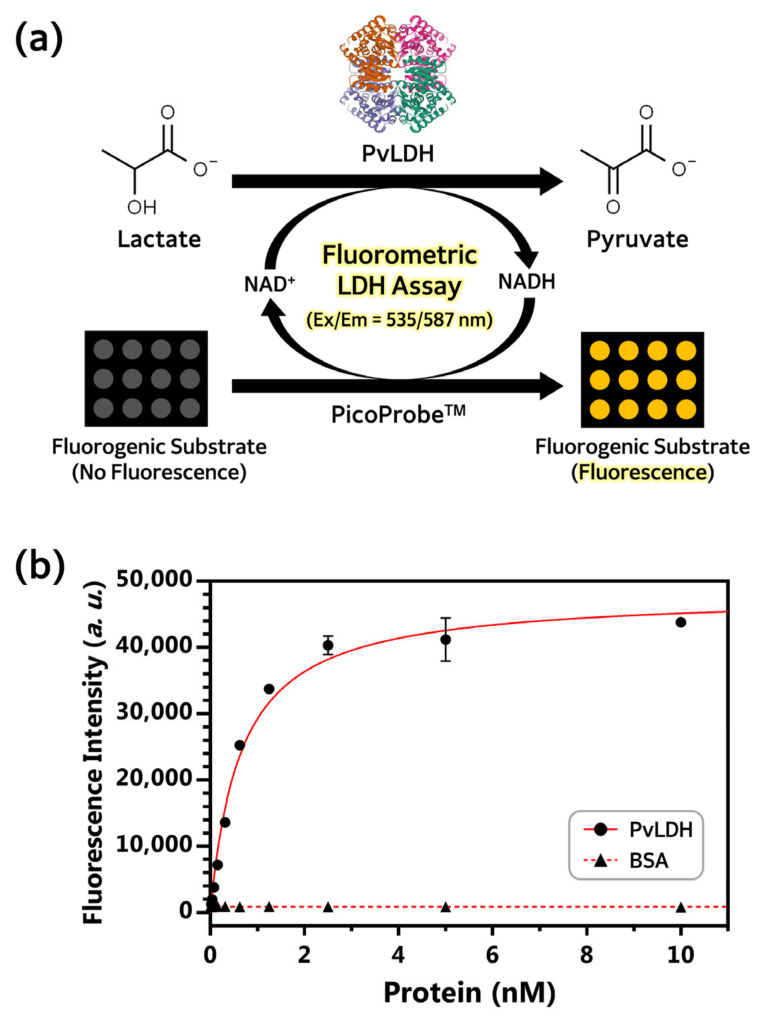
**Measurement of PvLDH enzyme activity.** (**a**) Schematic illustration of the principle for the analysis of enzyme activity of PvLDH. Fluorescence intensity was measured at 587 nm by exciting at 535 nm according to PvLDH concentration at a fixed substrate concentration. The image of the three-dimensional structure of PvLDH was adapted from Protein Data Band (ID: 5HS4). (**b**) PvLDH was serially diluted by ½ from 10 nM solution to obtain 11 solutions with different PvLDH concentrations, including that of 0 nM PvLDH concentration. BSA, a negative control, was included in the same concentration range as PvLDH. The total volume of sample per well was 100 μL, and the reaction was conducted at 37 °C for 30 min. The results were obtained from triplicate measurements. A black plate was used for fluorescence measurement. *a. u.*, arbitrary units; PvLDH, *P. vivax* lactate dehydrogenase; BSA, bovine serum albumin.

Finally, the enzymatic activity of PvLDH to degrade lactate was confirmed with an enzymatic assay using a fluorogenic substrate. This fluorescence-based LDH assay is based on the fact that LDH converts lactate to pyruvate and NAD^+^ to NADH, and the formed NADH acts on the PicoProbe^TM^ to generate a fluorescence signal (Ex/Em = 535/587 nm) from a fluorogenic substrate (Figure 8a). Several studies have reported successful monitoring LDH activity by this assay method [49,50,51]. The concentration of PvLDH was serially diluted by one-half from 10 nM. The serially diluted LDH solutions were mixed with LDH substrate and fluorogenic substrate at a fixed concentration of 100 µL volume. Fluorescence intensity was measured after the reaction was allowed to proceed on a black plate at 37 °C for 30 min. The fluorescence intensity increased with the PvLDH concentration (Figure 8b). Compared with the results for bovine serum albumin (BSA) under the same experimental conditions, PvLDH has sufficient enzyme activity to degrade lactate. Taken together, the three-dimensional structure of PvLDH purified after expression from *E. coli* Rosetta(DE3) and its enzymatic function of degrading lactate were both well maintained.

Considering the above results, we confirmed that functional PvLDH can be obtained in a high yield from a bacterial expression system. This means it can be used as a useful tool for several studies of *P. vivax*-mediated malaria. First, it can be used for the development of drug candidates against *P. vivax*-specific malaria. Recently, several studies have reported that plasmodial LDH is a useful target for antimalarial drugs [52,53,54]. Thus, *P. vivax*-specific antimalarial drugs, that are different from *P. falciparum* specific can be developed. Second, it can be used for the development of bioreceptors such monoclonal antibodies and aptamers for the diagnosis of *P. vivax*-specific malaria [32,55]. As mentioned above, since the life cycle of *P. vivax* in the human host is different that of *P. falciparum* [13,14,15,16,17,18], the parasite must be distinguished. Finally, it can be used as a useful tool for basic research related to identification of the metabolic pathway of parasites involved in PvLDH. Since PvLDH is essential for the development, division, and reproduction of the parasites, a complete understanding of PvLDH-related mechanism can provide us with useful diagnostic and clinical clues in the fight against *P. vivax* mediated malaria.

## 3. Materials and Methods

### 3.1. Cloning PvLDH Coding Gene and E. coli Rosetta(DE3) Strain Transformation

A 948 bp *PvLDH* gene (GeneBank: KX885923.1) was designed into the pUC-IDT plasmid. The pUC-IDT-*PvLDH* plasmid was synthesized by Integrated DNA Technologies (Coralville, IA, USA). The *PvLDH* gene was PCR amplified using the forward primer (5′-GGA TCC ATG ACG CCG AAA C-3′), reverse primer (5′-CTC GAG AAT GAG CGC CTT CA-3′), and nTaq-HOT polymerase (Enzynomics, Daejeon, Republic of Korea). The conditions for PCR amplification were: initial denaturation at 95 °C for 10 min, followed by 35 cycles of 30 s at 95 °C, 60 s at 55 °C, and 60 s at 72 °C, and final elongation for 5 min at 72 °C. PCR products were analyzed through electrophoresis using 1.0% agarose gel. The PCR product (1 μg) and pET-21a(+) (1 μg) were digested using *Bam*HI (20 units) and *Xho*I (20 units) (New England BioLabs, NEB, Ipswich, MA, USA) and ligated using 1 unit of T4 ligase (NEB). The *E. coli* Rosetta(DE3) strain was transformed using this recombinant plasmid pET-21a(+)-*PvLDH*. The genotype of Rosetta(DE3) used in this experiment was F^−^ *omp*T *hsdS*_B_(r_B_^−^ m_B_^−^) *gal dcm* (DE3) pRARE (Cam^R^). For transformation, the recombinant plasmid (1 ng) was dispensed into 100 μL of competent Rosetta(DE3) strain cells (Enzynomics) and slowly mixed and incubated on ice for 30 min. Subsequently, heat shock was applied at 42 °C for 1 min, and the cells were again cooled on ice. After adding 400 µL of super optimal broth with catabolite repression medium to the stabilized Rosetta(DE3) competent cells, they were cultured in a shaking incubator at 37 °C at 200 rpm for 1 h. These transformed Rosetta(DE3) cells were spread on a LB agar plate containing 100 µg/mL of ampicillin and incubated at 37 °C for 16 h. The colonies were collected and cultured in LB broth containing the same concentration of ampicillin, followed by extraction and purification of plasmids. The purified plasmids were digested with restriction enzymes (20 units of *Bam*HI and *Xho*I, respectively) to confirm whether the *PvLDH* gene was inserted into the vector. Finally, the DNA sequence of *PvLDH* coding gene was confirmed using a 3730xl DNA analyzer (Applied Biosystems, Waltham, MA, USA).

### 3.2. Optimizing PvLDH Expression

To optimize PvLDH expression in the transformed Rosetta(DE3) strain containing pET-21a(+)-*PvLDH* plasmid, cells were seeded in 20 mL of LB broth containing 100 µg/mL of ampicillin. The bacterial culture was incubated for 16 h in a shaking incubator at 37 °C and 200 rpm. The Rosetta(DE3) strain was cultured in 200 mL of LB broth containing 100 μg/mL of ampicillin until 0.7 optical density (OD) at 600 nm. The OD values were measured using OPTIZEN (Daejeon, Republic of Korea) UV−Vis spectrophotometer. Induction was performed at various temperatures, inducer concentrations, and induction times. First, induction temperature conditions were divided into low- and high-temperature overexpression at 20 °C for 16 h and 30 °C for 4 h, respectively. The concentrations of IPTG, the inducer, were set at 0, 0.001, 0.005, 0.01, 0.05, 0.1, 0.5, and 1 mM, respectively. The induction was completed for each condition and the Rosetta(DE3) strain was centrifuged at 13,000 rpm for 3 min. The obtained pellets were resuspended in a lysis buffer. A 12% polyacrylamide gel was used under reducing conditions to confirm the expression pattern of PvLDH according to induction conditions.

### 3.3. PvLDH Isolation and Purification

To overexpress PvLDH, the transformed Rosetta(DE3) strain was cultured until it reached 0.7 of OD_600_, following the conditions elucidated in Section 3.2. The bacterial culture was then centrifuged at 4 °C and 7500 rpm for 45 min, and the resulting pellets were resuspended in equilibrium buffer (pH 8.0; 500 mM NaCl, 50 mM KH_2_PO_4_, and 5 mM imidazole) supplemented with 100× protease inhibitor cocktail (GenDEPOT, Barker, TX, USA), and lysozyme (Sigma, Saint Louis, MO, USA). To lyse the cells and release the protein, the resuspended Rosetta(DE3) strain was homogenized using an ultrasonic processor (Sonics & Materials, VCX 130, Newtown, CT, USA) for 30 min at 30% amplitude, and sonicated for 10 s followed by a 20 s resting period. The homogenized cells were centrifuged at 9000 rpm for 30 min at 4 °C. The samples were then prepared for gravity flow affinity chromatography by passing the supernatant through a 0.22 µm syringe-driven filter from GVS (Bologna, Italy). Gravity flow affinity chromatography was performed using Ni-NTA resin to purify the PvLDH. To purify the 6× His-tagged PvLDH, Ni-NTA resin (Merck Millipore, Middlesex County, MA, USA) was packed into an empty column and equilibrated. The prepared sample was then passed through the column to allow the resin to bind to the protein. After washing the column with a washing buffer (pH 8.0; 500 mM NaCl, 50 mM KH_2_PO_4_, and 50 mM imidazole) to remove unbound proteins, the 6× His-tagged PvLDH was eluted with elution buffer (pH 8.0; 500 mM NaCl, 50 mM KH_2_PO_4_, and 500 mM imidazole). To achieve further purification, a series of dialysis steps was performed. The first dialysis was carried out using PBS (pH 7.4) containing 50 mM imidazole at 4 °C for 16 h. The second dialysis was then performed under similar conditions, but without the imidazole, to obtain the purified PvLDH. To ensure the removal of any remaining impurities, the solution containing PvLDH after dialysis was filtered using a 0.22 µm syringe-driven filter. This filtration step helped to improve the purity of the final protein sample.

### 3.4. Confirmation of Purity and PvLDH Tetramer Formation

The concentration of purified PvLDH was determined using a BCA protein assay (Thermo Fisher Scientific, Waltham, MA, USA) by measuring the absorbance at 562 nm. A standard curve of BSA was plotted for comparison. The PvLDH concentration was also confirmed using absorbance at 280 nm with a NanoDrop One^C^ spectrophotometer (Thermo Fisher Scientific). To assess the purity and molecular weight of the purified PvLDH, SDS-PAGE was performed. Samples were boiled at 100 °C for 10 min using 4× sample buffer (250 mM Tris-HCl (pH 6.8), 8% SDS, 40% glycerol, 8% β-mercaptoethanol, and 0.02% bromophenol blue) for loading PvLDH. PvLDH 2 μg was loaded onto a 5% stacking gel and a 12% separating gel. To identify the formation of PvLDH tetramers under native conditions, 2 μg of PvLDH was loaded onto a 5% stacking gel and a 10% separating gel and subjected to PAGE under non-reducing conditions. A 4× sample buffer (250 mM Tris-HCl (pH 8.8), 40% glycerol, and 0.02% bromophenol blue) was used for loading, and the samples were not boiled prior to electrophoresis. After electrophoresis, Commassie Brilliant Blue R-250 staining was performed to determine the molecular weight and purity of PvLDH by comparing its migration pattern to a protein ladder.

### 3.5. Analysis of PvLDH Activity

Enzymatic activity of the purified PvLDH was determined with a fluorometric assay kit (Abcam, Cambridge, UK) following the manufacturer’s protocol. Briefly, 100 µL of assay mixture per sample was added to each well of black microplate and the reaction was performed at 37 °C for 30 min. The assay mixture consisted of 45.5 µL of LDH assay buffer, 2.5 µL of the PicoProbe^TM^, 2 µL of substrate, and 50 µL of PvLDH at various concentrations. At this time, PvLDH solutions were prepared at various concentrations (0, 0.02, 0.04, 0.08, 0.16, 0.31, 0.63, 1.25, 2.50, 5.00, 10.00 nM) using two-fold serial dilution. The reaction was performed in triplicate at these 11 different concentrations. BSA was used as a negative control and reacted under the same conditions. Finally, fluorescence intensity was measured using a Cytation3 multimode microplate reader (BioTek, Winooski, VT, USA). The excitation and emission wavelengths were 535 and 587 nm, respectively. Based on the measured fluorescence intensity values, a dose–response curve was prepared using GraphPad Prism 7.0.

## 4. Conclusions

In this study, we constructed a transformed bacterial strain by inserting the *PvLDH* gene into pET-21a(+), without codon optimization. We introduced this recombinant plasmid DNA into the *E. coli* Rosetta(DE3) strain, which is suitable for eukaryotic protein expression. This transformed Rosetta(DE3) strain was used to optimize the conditions for PvLDH overexpression. The optimal conditions for inducing PvLDH were at 20 °C for 16 h using 0.1 mM IPTG. The use of minimal amounts of IPTG is advantageous for low-cost mass production of PvLDH. The PvLDH obtained in this study was in soluble form and was successfully purified with high purity (>95%) through immobilized-metal affinity chromatography. The yield was 31.0 mg/L, which is a considerable amount of PvLDH compared to that obtained in prior studies with the use of other bacterial strains. Furthermore, it was confirmed that the purified PvLDH has a tetrameric structure and enzymatic function, which are inherent characteristics of LDH. Therefore, PvLDH cloned, expressed, and purified from transformed bacteria without codon optimization was successfully demonstrated to exhibit the inherent structure and function of LDH. These findings provide valuable insights for conducting research on the mechanism of infectious diseases, parasite metabolism, screening for antimalarial drugs, and development of malaria diagnostics-based on *P. vivax* LDH.

## Data Availability

Data are contained within the article.
